# Dissemination of Mitochondrial DNA Variants: Looking at the ‘Bigger’ Picture of the Tumour Microenvironment in Rectal Cancer Patients

**DOI:** 10.1002/jex2.70097

**Published:** 2025-10-30

**Authors:** Kine Mari Bakke, Tonje Bjørnetrø, Paula A. Bousquet, Adriana M. Sanabria, Sebastian Meltzer, Torben Lüders, Anne‐Marie Siebke Trøseid, Espen Stang, Anne Negård, Elin Agathe Frøyen, Aida Kapic Lunder, Lars Gustav Lyckander, Hans Christian D. Aass, Kathrine Røe Redalen, Anne Hansen Ree

**Affiliations:** ^1^ Department of Oncology Akershus University Hospital Lørenskog Norway; ^2^ Department of Physics and Computational Radiology, Clinic for Radiology and Nuclear Medicine Oslo University Hospital Oslo Norway; ^3^ Department of Clinical Molecular Biology Akershus University Hospital Lørenskog Norway; ^4^ Institute of Clinical Medicine University of Oslo Oslo Norway; ^5^ Department of Biochemistry Oslo University Hospital Ullevål Oslo Norway; ^6^ Department of Pathology Oslo University Hospital Rikshospitalet Oslo Norway; ^7^ Department of Radiology Akershus University Hospital Lørenskog Norway; ^8^ Department of Pathology Akershus University Hospital Lørenskog Norway; ^9^ Department of Medical Biochemistry Oslo University Hospital Oslo Norway; ^10^ Department of Physics Norwegian University of Science and Technology Trondheim Norway

**Keywords:** cell density, extracellular vesicles, mitochondrial DNA, rectal cancer, tumour diffusion

## Abstract

The tumour microenvironment (TME) constitution is decisive for cancer outcome and is manifested in diffusion‐weighted (DW) magnetic resonance imaging (MRI). We hypothesized that the TME metabolic state is reflected by mitochondrial DNA (mtDNA) secreted in extracellular vesicles (EVs) and examined whether plasma EV‐mtDNA variants may divulge MRI‐assessed TME attributes of rectal cancer aggressiveness. On the diagnostic MRI scans from 60 rectal cancer patients, the apparent diffusion coefficient (ADC) was calculated on DW images (*n* = 29), and tumour volume (*n* = 57) and extramural vascular invasion (EMVI; all patients) were determined on anatomical images. Plasma EVs (all patients) were isolated by size exclusion chromatography and verified for EV features. The EV‐mtDNA was sequenced along with mtDNA in whole blood (WB; normal tissue) to calculate the EV/WB‐mtDNA total variant number (TVN) and heteroplasmic variant number (HVN)—as a proxy for TME intracellular mtDNA variants expelled in EVs. Low EV/WB‐mtDNA TVN and HVN, indicative of hampered clearance of mutated mtDNA via EVs, were associated with low ADC (high TME cell density; *p* = 0.018, *p* = 0.005) and a large tumour volume (*p* = 0.002, *p* = 0.003). Likewise, low EV/WB‐mtDNA TVN and HVN were associated with positive EMVI (tumour infiltration in blood vessels; *p* = 0.002, *p* = 0.003) and histologic ypN stage 1–2 (lymph nodes with tumour cells surviving radiotherapy; *p* = 0.002, *p* = 0.005), both indicators of high tumour aggressiveness. High cellular density may hamper the clearance of pathogenic tumour mtDNA variants by EVs and thus promote rectal cancer aggressiveness.

**Trial Registration**: ClinicalTrials.gov: NCT01816607. Registered 22 March 2013, https://clinicaltrials.gov/ct2/show/NCT01816607

## 1 Introduction

Colorectal cancer is a heterogenous disease characterized by the accumulation of mutations and a disordered immune system (Picard et al. 2020; Lichtenstern et al. 2020). The tumour microenvironment (TME) immune cell types, functional orientation, density and localization can determine the patient outcome (Pagès et al. 2018; Anitei et al. 2014), where circulating tumour DNA may provide complementary information (Li et al. 2022; Morais et al. 2022).

The deregulation of cellular metabolism is now recognized as a fundamental hallmark of cancer (Hanahan 2022). Tumours’ bioenergetic processes are altered and may result from pre‐existing or de novo mitochondrial DNA (mtDNA) mutations (Kopinski et al. 2021). mtDNA variants and mutations can coexist with wild‐type copies, a phenomenon termed heteroplasmy, which is considered an intermediate state between fixation or elimination of the acquired mtDNA mutations (Kopinski et al. 2021; Pérez‐Amado et al. 2021). It is suggested that mtDNA mutations causing adaptive advantages to tumour growth and invasion are enriched in malignant cells, and recent studies show heteroplasmy shifting as a potential shaper of tumour progression and treatment response (Pérez‐Amado et al. 2021).

Extracellular vesicles (EVs) are nanosized lipid membrane vesicles secreted by cells for cell‐to‐cell and long‐distance communication as well as clearance of cellular material. In cancer, EVs secreted from tumour‐ or nonmalignant cells can contribute to tumour progression and therapy resistance (Bao et al. 2021; Möller and Lobb 2020). The EV cargo is heterogeneous and reflects the cell of origin. The vesicles contain a variety of proteins, lipids and nucleic acids (Bao et al. 2021). The packaging of DNA into vesicles protects it from the external environment and recognition by the immune system. The biological function of EV‐DNA is still largely unknown, but it may play a role in the maintenance of cellular homeostasis and intercellular messaging (Elzanowska et al. 2021). EVs have been shown to contain mitochondrial components such as mtDNA, and are proposed as a transfer mechanism for altered mitochondrial constituents (Elzanowska et al. 2021; Liu et al. 2020).

Diffusion‐weighted (DW) magnetic resonance imaging (MRI) measures the free diffusion of water molecules in a tissue. As water molecules are inhibited in their free diffusion by cell membranes, DW‐MRI reflects the cell density of the tissue. This is widely acknowledged due to the high cell density of solid tumours being easily recognizable by DW‐MRI (Messina et al. 2020). From a DW image series with increasing diffusion weighting, it is possible to calculate the apparent diffusion coefficient (ADC), a quantitative measure of the tissue diffusion. A low ADC reflects high cell density and has been shown to be associated with a more aggressive tumour profile in several pelvic malignancies, including rectal cancer (Bollineni et al. 2015).

The secretion of EVs is a regulated process induced by various stimuli or pathological situations. Physical contact with neighbouring cells provokes EV generation and release (van Niel et al. 2022). In this rectal cancer study, we investigated whether the EV‐mtDNA cargo, possibly released from the tumour cells, might be dependent on physical tumour properties. We showed that MRI‐measured tumour ADC and volume were associated with the mtDNA cargo of EVs, which further was associated with the presence or absence of extramural vascular invasion (EMVI; the tumour infiltration in blood vessels just outside the bowel wall at the time of diagnosis) and how advanced was the histopathologic tumour stage in the surgical specimens, each a risk indicator of treatment outcome and metastatic behaviour.

## 2 Materials and Methods

### 2.1 Ethics Approval and Consent to Participate

The prospective biomarker study OxyTarget in rectal cancer (NCT01816607), undertaken at Akershus University Hospital (Lørenskog, Norway), was approved by the Institutional Review Board and Regional Committee for Medical and Health Research Ethics of South‐East Norway (reference number REK 2013/152) and conducted in accordance with the Helsinki Declaration. Written informed consent was required to participate.

### 2.2 Patients and Procedures

The total study cohort presented here consists of 60 patients with verified rectal adenocarcinoma (characteristics in Table S1) who were treated according to the prevailing national guidelines with neoadjuvant oncologic treatment, consisting of long‐course chemoradiotherapy or short‐course radiotherapy, followed by surgery (*n* = 49, of whom two declined surgery). Eleven patients had direct surgery. Five of the 60 study participants had metastatic disease at the time of diagnosis and additionally received palliative chemotherapy.

At the time of diagnosis (baseline), patients had radiologic disease staging according to the TNM classification system (Sobin and Compton 2010), with the tumour extension within the pelvic cavity determined by MRI and the metastatic status determined by computed tomography of the thoracic and abdominal cavities. MRI‐measured EMVI was coded as negative or positive (Smith et al. 2008); positive EMVI was defined as tumour signal within the lumen of blood vessels with irregular walls, continuously extending from the primary tumour to outside the bowel wall. Of the 60 patients, 39 also had a DW‐MRI sequence with *b* values 0, 25, 50, 100, 500 and 1000 s/mm^2^, to estimate the ADC (Bakke et al. 2020). For 57 patients, the high‐resolution T2‐weighted images enabled estimation of whole‐tumour volume after delineation by experienced radiologists (Bakke et al. 2020). The delineation was co‐registered onto the DW images and used to estimate the median tumour ADC. The resected primary tumour specimens were histologically evaluated according to standard criteria—ypTN‐status for patients who had received neoadjuvant oncologic treatment and pTN‐status for those who had proceeded directly to surgery.

### 2.3 Blood Sample Processing

Research blood samples were collected at the time of study inclusion, on the same day that MRI scans were conducted for all patients. Whole blood (WB) was collected by venipuncture in PAXgene RNA tubes (PreAnalytiX GmbH, Hombrechtikon, Switzerland) and stored at −80°C. Prior to DNA extraction, 150 µL of the PAXgene WB samples was transferred to microcentrifuge tubes and centrifuged at 5000 *g* for 10 min before the supernatants were carefully removed. Sodium citrate‐treated BD Vacutainer CPT tubes (Becton, Dickinson and Company, Franklin Lakes, NJ, USA) were used for preparation of plasma and peripheral blood mononuclear cells (PBMCs). The citrate plasma samples were prepared by centrifugation at 2000 *g* for 10 min, and aliquots were stored at −80°C. Circulating EVs were isolated from 100‐µL plasma by qEV single size exclusion chromatography columns (IZON Science, Oxford, UK; collected as 500‐µL EV fraction) and proteinase‐ and DNase‐treated (Qiagen, Hilden, Germany and DNaseI Amplification Grade; Sigma‐Aldrich, Saint Louis, MO, USA) prior to DNA analysis. The detailed isolation procedure and EV characterization with Western blot analysis (expression of CD9, CD63, ALIX and APOA1 and the absence of GM130), Nanoparticle Tracking Analysis (NTA) and transmission electron microscopy (TEM) were recently reported (Bjørnetrø et al. 2023). B‐cells, CD4 T‐cells, CD8 T‐cells and monocytes were sorted from PBMCs. The PBMCs were prepared from 6 to 8 mL of WB centrifuged with a horizontal rotor centrifuge at 1500 *g* for 20 min. The buffy coat layer was transferred to a fresh 15‐mL tube, resuspended and washed twice in phosphate‐buffered saline (Gibco by Life Technologies, Paisley, UK) with centrifugations at 300 *g* for 15 and 10 min. The mononuclear cells were thereafter resuspended in RPMI‐1640 medium (Gibco) supplemented with 10% dimethyl sulfoxide (Sigma‐Aldrich, Saint Louis, MO, USA) and immediately frozen at −150°C. The PBMCs were thawed and sorted on BD FACSAria, as detailed in Supporting Material. DNA was isolated from an average of 136,900 cells (range: 3500–434,000).

### 2.4 mtDNA Sequencing

We have previously sequenced mtDNA from selected WB samples (*n* = 44) and plasma EV samples (*n* = 8) (Bjørnetrø et al. 2023; Bousquet et al. 2022). With the additional mtDNA sequencing of EVs and WB samples for the current analysis, the total cohort comprised 60 patients. Table S2 delineates the sequence depth of each EV‐ and WB‐mtDNA sample. The ratio of EV‐to‐WB (EV/WB) mtDNA variants was calculated for each patient. To summarize the sequencing procedure (Bjørnetrø et al. 2023), total DNA was extracted using the DNeasy Blood & Tissue Kit (Qiagen), and the DNA was quantified by Qubit fluorometer 2.0 (Thermo Fisher Scientific, Waltham, MA, USA). The mtDNA was amplified in two long‐range targeted PCRs, spanning the complete human mitochondrial genome (16,569 bases long). The library construction followed the suggested protocols of Human mtDNA Genome for the Illumina Sequencing platform (Illumina Inc., San Diego, CA, USA) but was adapted with a temperature gradient (51°C –68°C) during the first PCR amplification. The DNA was subsequently purified using gel electrophoresis, and bands representing the mtDNA amplicons (9.1 and 11.2 kilobases of length) were cut out from the gel. Extraction and quantification of the mtDNA were performed using QIAEX II Gel Extraction Kit (Qiagen) and Qubit dsDNA HS Assay Kit (Thermo Fisher Scientific). The mitochondrial genomes were sequenced using the Nextera XT DNA Library Preparation Kit and Nextera XT Index Kit (both Illumina). The full mtDNA was successfully sequenced on a MiSeq Benchtop Sequencer (Illumina) using a MiSeq Reagent Kit v3 (Illumina) with 2 × 300‐basepair read lengths.

### 2.5 Sequence Analysis

The mtDNA was mapped to the revised Cambridge Reference Sequence (GenBank ID NC_012920.1), the fully corrected version of the original human mtDNA sequence (Anderson et al. 1981; Andrews et al. 1999) using the MiSeq Reporter built‐in software v2.6 (Illumina). The Mutserve via mtDNA‐Server (https://mtdna‐server.uibk.ac.at) (Weissensteiner et al. 2016) was used for variant calling and annotation with default parameters and filter settings; minimum base call quality score for a call (<30), Indel repeat length (>8) and low variant frequency (<0.010). Only variants with a final filter pass were included for downstream analysis. This variant caller was shown to have the best performance compared to other variant callers with regard to evaluating heteroplasmy (Ip et al. 2022). In accordance with this, variant frequencies <0.990 were defined as heteroplasmy (comprising all types of variants: recent germline mutations, somatic mutations and ancient adaptive polymorphisms). The Ensembl Variant Effect Predictor software (Mclaren et al. 2016) was used with default parameters to predict the potential role of the variants. High/moderate effects on protein function were defined as missense, stop gained, stop lost, start lost and low‐impact variants as synonymous, coding sequence, stop retained, incomplete terminal codon, as guided by The Ensembl Variant Effect Predictor.

### 2.6 Statistical Considerations

Analyses were performed using GraphPad Prism v9.2.0 and SPSS v.28. Differences between two groups were analysed by paired Student's *t*‐test when comparing paired samples from patients and Mann–Whitney *U* test when comparing differences between patients. Repeated measures one‐way ANOVA and Tukey's multiple comparisons test were used to compare more than two groups. For continuous data, Spearman's correlation analysis was performed. *p* values less than 0.05 were considered statistically significant.

## 3 Results

### 3.1 Plasma EVs Contain Mutated mtDNA

**FIGURE 1:**
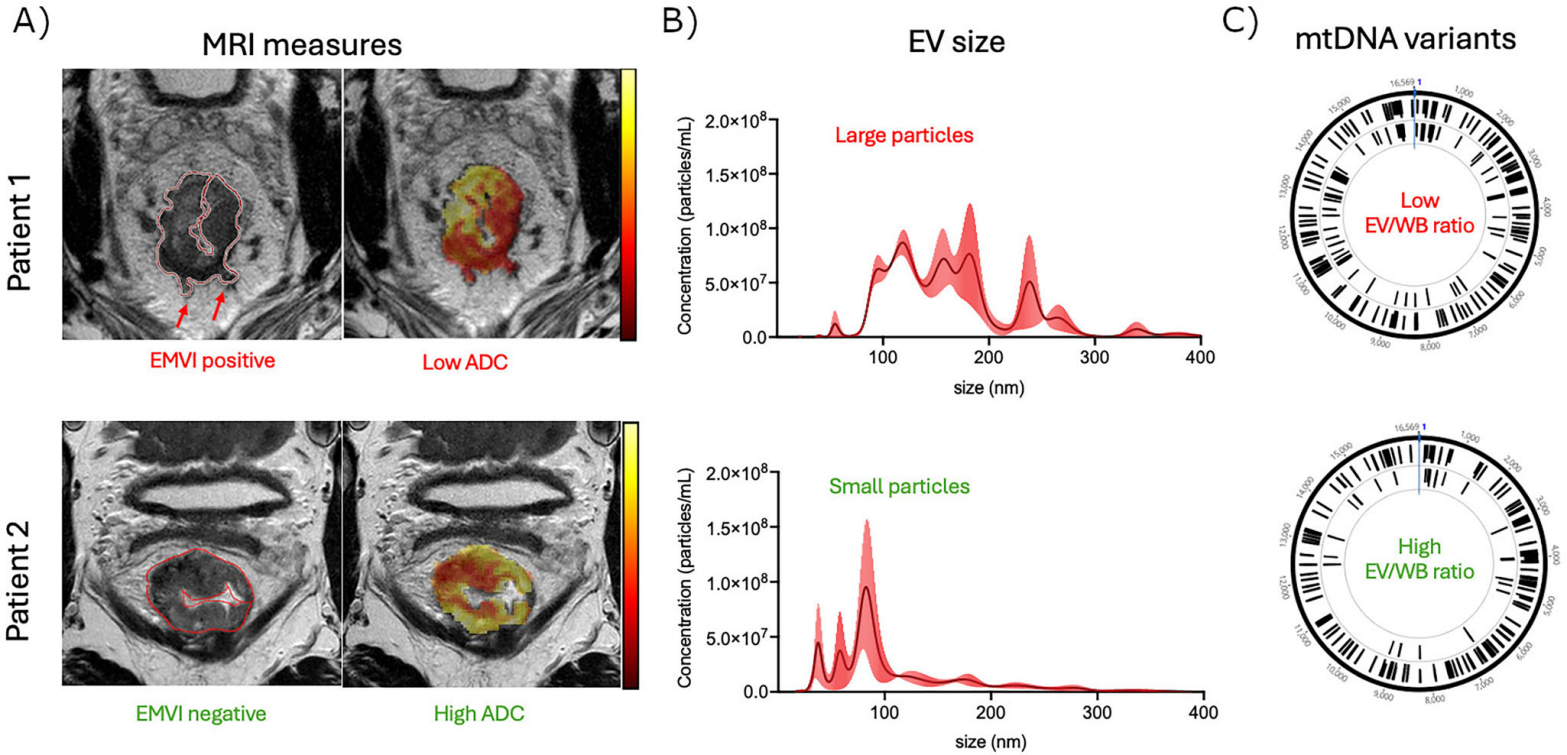
Tumour and plasma extracellular vesicles (EVs) characteristics in two rectal cancer patients. (A) Magnetic resonance imaging (MRI) measures; The left images show T2‐weighted MRIs with the tumours delineated in red; the extramural vascular invasion (EMVI) site is indicated on the top image. The right images show the apparent diffusion coefficient (ADC) maps of the tumours as overlays, where yellow marks higher ADC. (B) EV size; histograms of Nanoparticle Tracking Analysis show the respective EV sizes. (C) Mitochondrial DNA (mtDNA) variants; circular representation of the mtDNA sequence data shows variants detected in the EVs (outer circles) and the corresponding whole blood (WB; inner circles).

Isolated plasma EVs from this cohort of 60 rectal cancer patients have been characterized previously by NTA, TEM and Western blot analysis (presence of CD63, CD9, ALIX and APOA1 and the absence of GM130) and shown to contain mutated mtDNA (Bjørnetrø et al. 2023). Here, we explored the EVs and mtDNA cargo with regard to clinical and experimental TME features. Figure 1 summarizes the selected parameters on baseline MRI (A) together with plasma EV (B) and mtDNA (C) characteristics from two of the patients, one with MRI‐defined high‐risk (upper panel; red letters) and the other with low‐risk (lower panel; green letters) disease.

**FIGURE 2:**
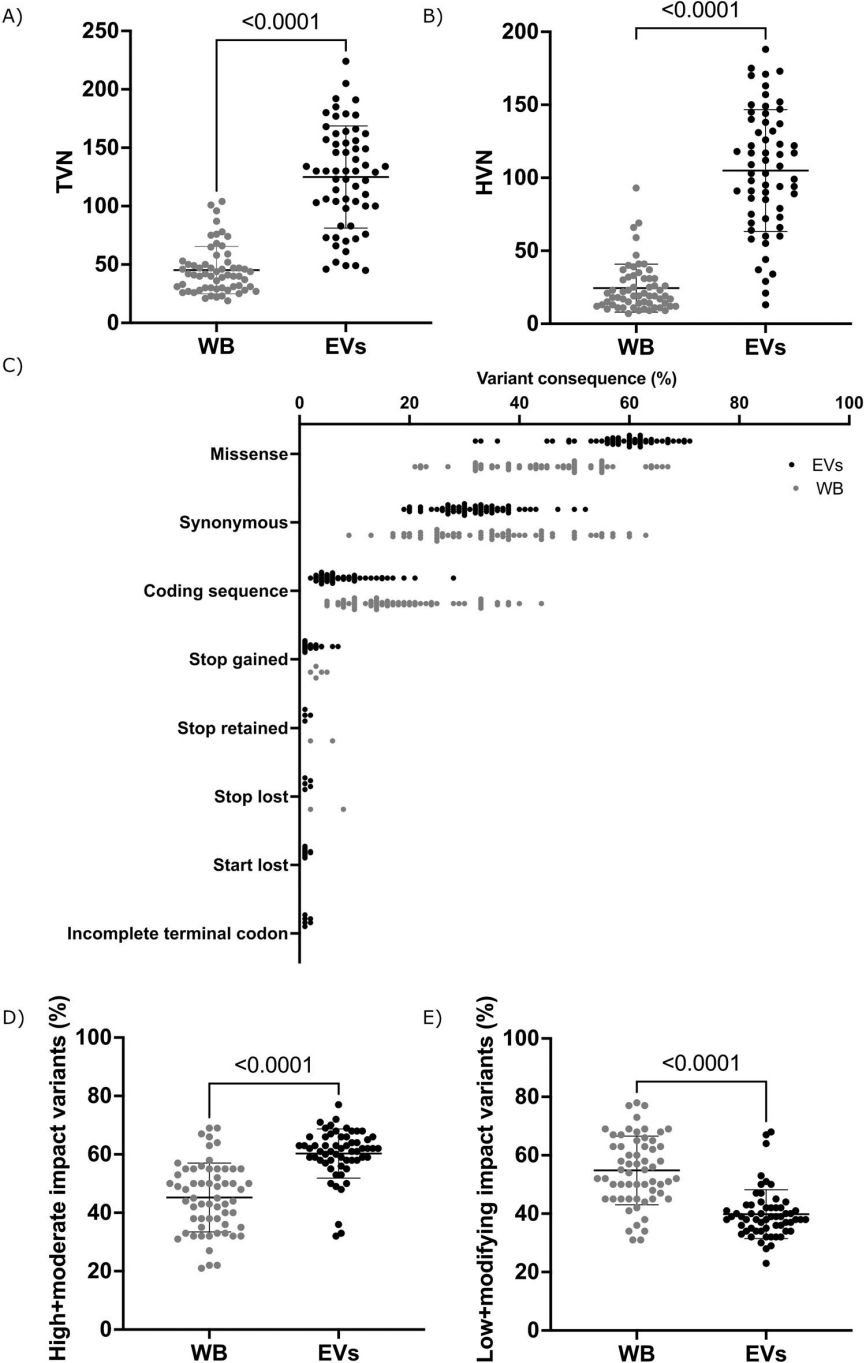
Mitochondrial DNA (mtDNA) in paired samples of whole blood (WB) and plasma extracellular vesicles (EVs) from rectal cancer patients (*n* = 60). The mtDNA (A) total variant number (TVN) and (B) heteroplasmic variant number (HVN; frequency < 0.990). (C) Predicted variant effects of each EV‐mtDNA and WB‐mtDNA sample. The percentage of mtDNA variants with predicted (D) high/moderate effects on protein function (missense, stop gained, stop lost, start lost) and (E) low‐impact effects on protein function (synonymous, coding sequence, stop retained, incomplete terminal codon). All comparisons were calculated with a paired Student's *t*‐test.

Here, to comprise all 60 study patients, we sequenced the complete mitochondrial genome encapsulated in plasma EVs to characterize the cell‐free but protected circulating mtDNA and compared it with the paired normal tissue intracellular WB‐mtDNA. Five patients also had their PBMC B‐cells, CD4 T‐cells, CD8 T‐cells and monocytes sequenced for comparison of TVN and HVN to WB, with no differences (TVN; *p* = 0.18, HVN; *p* = 0.19; Figure S1). Confirming our previous data in the smaller sample set (Bjørnetrø et al. 2023), EVs contained significantly higher mtDNA total variant number (TVN; *p* < 0.0001; Figure 2A) and heteroplasmic variant number (HVN; *p* < 0.0001; Figure 2B) than WB. The number of EV‐mtDNA variants was increased—some of which were not detected in the paired WB samples—and these variants were predicted to have high/moderate effects on protein function (missense, stop gained, stop lost, start lost; *p* < 0.0001). In contrast, numbers were reduced for low‐impact EV‐mtDNA variants (synonymous, coding sequence, stop retained, incomplete terminal codon; P < 0.0001) when compared to WB‐mtDNA (Figure 2C–E). Of note, the TVN, HVN and variant effect prediction of mtDNA variants in EVs and WB did not seem to correlate (data not shown). Hence, EV/WB‐mtDNA variant scores were calculated, potentially representing additional mtDNA information from the tumour, and used in further analyses.

### 3.2 Associations Between Plasma EVs and MRI‐Assessed Tumour Diffusion and Volume

A variety of factors may contribute to tissue alterations in EV secretion and internalization, and we explored whether plasma EV size, concentration and mtDNA cargo might be related to TME attributes as determined by MRI (Table 1). The assessed parameters were tumour ADC (tumour diffusion) and tumour volume. No correlations were found between EV concentration (measured by NTA) and the MRI parameters, but patients with low tumour ADC, reflecting high cell density, had larger vesicles (measured by NTA) in the circulation (rho = −0.502, *p* = 0.001). Two cases were selected for EV evaluation using TEM and NTA (Figure S2) and showed sample heterogeneity, as expected (Bjørnetrø et al. 2023). As further shown in Table 1, tumour ADC correlated positively with the EV/WB‐mtDNA variant score—EVs had higher mtDNA TVN (rho = 0.378, *p* = 0.018) and HVN (rho = 0.444, *p* = 0.005) relative to the normal tissue WB, at lower TME cell density. Accordingly, the lower the ADC (higher TME cell density), the more comparable the number of mtDNA variants with high/moderate effects on the protein coding regions (rho = 0.431, *p* = 0.006) in the EVs and WB, suggesting less of these variants expelled in EVs; consequently, a negative correlation (for higher ADC) with EV‐mtDNA variants with low‐impact effects (rho = −0.432, *p* = 0.006) was seen. The tumour volume was inversely correlated with EV/WB‐mtDNA TVN and HVN (rho = −0.395, *p* = 0.002; rho = −0.390, *p* = 0.003), suggesting less mtDNA variants are expelled in circulating EVs from larger tumours. Taken together, tumour diffusion seemed to be linked to circulating EVs and detectable mutated EV‐mtDNA.

**TABLE 1:** Correlations of plasma EV‐ and EV/WB‐mtDNA parameters with MRI parameters.

MRI parameter		Plasma EV parameter				EV/WB mtDNA parameter							
		Size		Concentration		Ratio of TVN		Ratio of HVN		Ratio of variants with high/moderate function change		Ratio of variants with low‐impact function change	
	*n*	Rho	*p*	Rho	*p*	Rho	*p*	Rho	*p*	Rho	*p*	Rho	*p*
ADC (tumour diffusion)	39	**−0.502**	**0.001**	−0.078	0.64	**0.378**	**0.018**	**0.444**	**0.005**	**0.431**	**0.006**	**−0.432**	**0.006**
Tumour volume	57	0.236	0.077	−0.176	0.19	**−0.395**	**0.002**	**−0.390**	**0.003**	−0.198	0.14	0.185	0.17

Abbreviations: ADC, apparent diffusion coefficient; EV, extracellular vesicles; HVN, heteroplasmic variant number; MRI, magnetic resonance imaging; mtDNA, mitochondrial DNA; TVN, total variant number; WB, whole blood.

Bold values indicate *p* < 0.05

### 3.3 Associations Between Plasma EVs and Established Outcome Indicators

We further explored whether the EV size, concentration and mtDNA cargo might be related to the baseline EMVI score and histopathologic tumour stage of the surgical specimen (Table 2). Patients with positive tumour EMVI had larger plasma vesicles (*p* = 0.008) and EV mtDNA TVN and HVN comparable to WB; that is, lower EV/WB‐mtDNA TVN (*p* = 0.002) and HVN (*p* = 0.003) than EMVI‐negative cases, suggesting less mtDNA variants expelled in EVs from blood vessel‐infiltrating tumours, an MRI sign of incipient spread to distant organs. The TNM stage at the time of diagnosis was not associated with any of the plasma EV‐mtDNA parameters. Eleven of the 60 patients had direct surgery, and those with a more locally invasive primary tumour (pT stage 3–4) had lower EV/WB‐mtDNA HVN (*p* = 0.024) than those with the most early‐stage disease (pT 1–2). The remaining 49 subjects received neoadjuvant radiation before resection of the residual tumour, except for two patients who declined surgery. Those with involved lymph nodes in the surgical specimen (ypN stage 1–2), representing metastasizing tumour cells that had survived the neoadjuvant therapy, had at baseline presented with lower EV/WB‐mtDNA TVN (*p* = 0.002) and HVN (*p* = 0.005) compared to the patients who had the lymph nodes sterilized (ypN stage 0) by the neoadjuvant therapy. To summarize, we suggest that plasma EVs can act as containers for cells to clear out damaging mtDNA variants and propose that high‐diffusion tumours have a high ability to expel acquired mtDNA mutations in EVs. Conversely, in low‐diffusion tumours, EVs will more likely remain in the TME and be taken up by neighbouring cells, where damaging mtDNA variants can continue to support tumour growth.

**TABLE 2:** Associations of plasma EV‐ and EV/WB‐mtDNA parameters with routine assessment tumour parameters.

Tumour parameter			Plasma EV parameter				EV/WB‐mtDNA parameter							
			Size (nm)		Concentration (10^9^)		Ratio of TVN		Ratio of HVN		Ratio of variants with high/moderate function change		Ratio of variants with low‐impact function change	
		*n*	Median (range)	*p*	Median (range)	*p*	Median (range)	*p*	Median (range)	*p*	Median (range)	*p*	Median (range)	*p*
EMVI score	Neg	29	**109.2 (73.4‐203.3)**	**0.008**	4.40 (1.10–49.0)	0.45	**3.25 (1.07**–**7.35)**	**0.002**	**7.00 (0.84**–**15.7)**	**0.003**	1.37 (0.67–2.82)	0.23	0.67 (0.46–1.31)	0.17
	Pos	31	**129.1 (98.1‐368.0)**		4.00 (1.10–32.0)		**2.41 (0.63**–**6.39)**		**3.79 (0.59**–**16.7)**		1.24 (0.75–2.68)		0.78 (0.49–1.47)	
T stage	2–3	36	111.6 (73.4–242.9)	0.064	3.35 (1.10–49.0)	0.82	3.10 (0.82–7.35)	0.36	5.13 (0.71–16.7)	0.76	1.32 (0.67–2.82)	0.89	0.72 (0.46–1.31)	0.93
	4	24	125.5 (98.1–368.0)		4.35 (1.10–44.0)		2.75 (0.63–5.36)		4.54 (0.59–13.6)		1.31 (0.75–2.06)		0.71 (0.49–1.47)	
N stage	0	25	118.7 (73.4–159.3)	0.86	3.80 (1.10–22.0)	0.48	3.55 (0.82–6.21)	0.059	7.00 (0.71–13.6)	0.18	1.32 (0.67–2.68)	0.86	0.70 (0.46–1.47)	0.83
	1–2	35	116.2 (73.8–368.0)		5.60 (1.10–49.0)		2.74 (0.63–7.35)		4.12 (0.59–16.7)		1.32 (0.88–2.82)		0.72 (0.46–1.18)	
M stage	0	55	114.9 (73.4–368.0)	0.45	3.80 (1.10–49.0)	0.086	3.07 (0.63–7.35)	0.10	4.68 (0.59–16.7)	0.18	1.32 (0.67–2.82)	0.27	0.71 (0.46–1.47)	0.31
	1	5	129.1 (98.1–196.8)		6.60 (5.80–32.0)		1.73 (1.22–4.48)		2.35 (1.46–8.27)		1.24 (0.92–1.38)		0.76 (0.70–1.11)	
pT stage	1–2	3	106.0 (78.8–141.1)	0.99	2.70 (2.20–22.0)	0.15	4.05 (3.56–5.57)	0.13	**10.62 (8.47**–**12.0)**	**0.024**	1.57 (1.26–2.07)	0.19	0.60 (0.59–0.71)	0.19
	3‐4	8	104.7 (73.4–368.0)		2.05 (1.10–9.60)		3.12 (1.07–6.21)		**5.04 (0.84**–**8.73)**		1.32 (0.67–1.54)		0.75 (0.46–1.31)	
pN stage	0	5	106.0 (73.4–141.1)	0.93	2.60 (2.10–9.60)	0.27	3.26 (1.07–5.57)	0.79	7.00 (0.84–12.0)	0.79	1.07 (0.67–1.57)	0.082	0.93 (0.59–1.31)	0.18
	1–2	6	104.4 (73.8–368.0)		1.70 (1.10–22.0)		3.37 (2.65–6.21)		7.03 (3.79–10.62)		1.45 (1.31–2.07)		0.66 (0.46–0.78)	
ypT stage	0–2	17	113.3 (96.8–203.3)	0.38	3.90 (1.10–49.0)	0.47	3.04 (0.82–7.35)	0.30	4.82 (0.71–15.91)	0.28	1.37 (0.86–2.82)	0.35	0.67 (0.46–1.10)	0.36
	3–4	30	128.1 (97.5–255.9)		5.30 (1.40–44.0)		2.73 (0.63–6.39)		4.02 (0.59–12.21)		1.23 (0.75–2.68)		0.78 (0.49–1.47)	
ypN stage	0	29	113.3 (96.8–255.9)	0.065	4.10 (1.10–49.0.)	0.15	**3.17 (0.82**–**7.35)**	**0.002**	**4.68 (0.71**–**15.91)**	**0.005**	1.44 (0.86–2.81)	0.054	**0.67 (0.46**–**1.16)**	**0.024**
	1–2	18	131.4 (98.1–242.9)		6.85 (1.40–44.0.)		**2.06 (0.63**–**4.48)**		**2.83 (0.59**–**8.27)**		1.17 (0.75–1.97)		**0.83 (0.49**–**1.47)**	

Abbreviations: EMVI, extramural vascular invasion; EV, extracellular vesicles; HVN, heteroplasmic variant number; M, metastasis; mtDNA, mitochondrial DNA; N, node; p, histopathologic status after surgery alone; T, tumour; TVN, total variant number; WB, whole blood; yp, histopathologic status after neoadjuvant oncologic treatment and surgery.

## 4 Discussion

In this study, we verified that plasma EVs from rectal cancer patients contain mutated mtDNA (Bjørnetrø et al. 2023). We further assessed the number of mtDNA variants in EVs relative to that of the paired normal tissue intracellular WB—the EV/WB‐mtDNA variant score—as a liquid biopsy potentially reflecting tumour mtDNA variants expelled in EVs. We found that high EV/WB‐mtDNA TVN and HVN, comprised of variants with highly functional effects, were associated with high tumour diffusion (high ADC) and a small tumour volume on MRI, indicative of efficient tissue clearance of unfavourable mtDNA variants via circulating EVs by primary tumours at less risk of metastasizing. Likewise, lower EV/WB‐mtDNA TVN and HVN were associated with tumour infiltration in blood vessels at the time of diagnosis (positive EMVI) and lymph nodes with tumour cells surviving radiotherapy (ypN stage 1–2), both indicators of high tumour aggressiveness. Our findings support a postulation that the most pathogenic mtDNA variants in the primary tumour can be removed by EVs—if the physical constitution of the TME permits.

As many as 40%–50% of rectal cancer patients either present with or progress to metastatic disease. Established biomarkers of metastatic risk on the diagnostic MRI include low ADC (high density of tumour cells) and the presence of EMVI (incipient hematogenous spread to distant organs) (Siddiqui et al. 2017). Likewise, ypN stage 1–2 (lymph nodes with tumour cells surviving radiotherapy) in the surgical specimen is a biomarker of treatment‐resistant clonogenic tumour cells. Within this frame of reference, it is notable that EVs promote cell growth and survival and help shape the TME by increasing invasive and metastatic activities (Bao et al. 2021; Chang et al. 2021).

Circulating cell‐free DNA comprises nuclear as well as mtDNA, and depending on the mechanism of release, several structural forms have been identified—in particulate structures (EVs) or as macromolecular structures (nucleosomes, virtosomes, DNA traps or linked to serum proteins or cell‐free membrane elements) (Thierry et al. 2016). Cancer cells might gain functional mtDNA mutations as a biological strategy to adjust the energy metabolism during adaptation to oncogenic conditions (Pérez‐Amado et al. 2021; Filograna et al. 2021), but the detailed importance of mitochondrial components in EVs is currently incompletely known (reviewed in Liu et al. 2020). mtDNA has been found in the lumen or the external surface of vesicles from various cell types (Marcoux et al. 2019; Torralba et al. 2018; Guescini et al. 2010; Jeon et al. 2019; Guescini et al. 2010; Tsilioni and Theoharides 2018) and in circulating EVs from patients with metastatic breast cancer (Sansone et al. 2017), glioblastoma (Soltész et al. 2022) and hepatocellular carcinoma (Li et al. 2020). These studies further suggest mtDNA‐carrying EVs to be involved in intercellular communication, immune response modulation and stem cell management of oxidative stress. Here, we report that the mitogenome protected from DNase‐ and proteinase treatment of plasma EVs from rectal cancer patients is highly mutated with variants mainly in a heteroplasmic state. This is consistent with previous reports from cancer patients (Soltész et al. 2022; Li et al. 2020; Vikramdeo et al. 2022). Our study further supports the notion that the release of mtDNA in EVs is a regulated process in the TME and that the circulating EV‐mtDNA can originate from the tumour; however, both our own previous research (Bjørnetrø et al. 2023) and that by others (Li et al. 2020; Vikramdeo et al. 2023) indicate that only a limited amount of fixed tumour‐derived mtDNA variants can be detected in circulating EVs. Since the variants we detected here were predicted to be highly functional, it is tempting to speculate that the release of mtDNA by EVs might be involved in the regulation of tumour aggressiveness.

It has been suggested that EVs are a way for cells to eliminate DNA mutations known to accumulate during aging, and the decline in circulating EV‐mtDNA levels with age may reflect a defective clearance of cellular material (Lazo et al. 2021). Cancer cells secrete more EVs with altered composition compared to normal cells, and a variety of factors contribute to the secretion patterns (Bebelman et al. 2021). Our data suggested that tumours with low diffusion, reflecting high cell density, secreted EVs of larger size and had mtDNA more comparable to normal tissue (lower EV/WB‐mtDNA TVN and HVN). We propose that high‐diffusion tumours have a high ability to expel mutated mtDNA by EVs, and that in low‐diffusion tumours, the small EVs remain in the TME and are taken up by neighbouring cells in which damaging mtDNA variants continue to support tumour progression. Low ratio of EV‐to‐WB mtDNA at baseline reflects an adverse TME that is associated with the presence of EMVI and survival of metastasizing tumour cells (involved lymph nodes) after neoadjuvant treatment. Of note, as recently shown mechanistically, mtDNA enriched in small EVs secreted by colon cancer cells is transferred to adjacent normal epithelial cells, in which it initiates mitochondrial activation and promotes tumorigenesis (Guan et al. 2024). mtDNA‐containing EVs also promote breast cancer cell invasiveness (Rabas et al. 2021). Moreover, pathogenic mtDNA mutations from highly metastatic tumour cells are horizontally transferred to low‐metastatic tumour‐ and TME stromal cells, enhancing the metastatic potential (Takenaga et al. 2021). A recent discovery highlighted that tumour‐cell immune evasion may result from homoplasmic replacement in TME lymphocytes of mutant mtDNA transferred from the tumour cells (Ikeda et al. 2025). An ongoing study for early‐stage colorectal cancer patients (NCT02579278) investigates the presence or absence of circulating tumour DNA and the relationship to EMVI as a predictive biomarker of metastatic outcome. Perhaps plasma EV‐mtDNA might also be considered in future studies, although the analyses are labour‐intensive and time‐consuming.

We used MRI‐measured ADC as an approximation for cell density, while the ADC can be influenced by other measures, such as cell stiffness. For most of the study cases, the surgical tumour specimen had been through neoadjuvant treatment, permuting the TME and making tissue histology unapt in this setting. Tumour volumetry on MRI comes with analytical uncertainties, but each assessment was undertaken by two experienced radiologists with consensus (Bakke et al. 2020). The MRI‐diagnosed EMVI status has superior accuracy to current TNM staging in rectal cancer (Lord et al. 2022). Other limitations of this study involve the heterogeneity and origin of the collected EVs. We isolated bulk plasma EVs with a focus on a subset of small EVs but did not evaluate EV subtypes. NTA typically results in an overestimation of EV size (Welsh et al. 2024); however, the TEM analysis differentiated between samples. Further studies are needed to distinguish whether the findings are a result of prevented EV release or the promotion of EV internalization. Methodological issues of mtDNA analysis related to the freezing of WB samples were likely negligible, as the WB was collected in PAXgene RNA tubes specifically designed for long‐term storage of frozen specimens, and the RPMI‐1640 medium was used for the long‐term cryopreservation of PBMCs before a single thaw for analysis. The study of mtDNA heteroplasmy is challenging, especially the detection of low‐level heteroplasmy. Some bias from both the sequencing and bioinformatic tools is to be expected. Other limitations include a small sample size for statistical analysis and investigations only by associations.

In conclusion, using prospectively collected anatomical and DW‐MRI data and a matched plasma biobank from rectal cancer patients, we demonstrated by associations that plasma EVs contained mutated mtDNA that might represent cleared pathogenic mtDNA variants from the TME, likely crucial for the patient outcome.

## Author Contributions

**Kine Mari Bakke**: conceptualization, formal analysis, validation, investigation, visualization, methodology, writing – original draft, project administration, writing – review and editing. **Tonje Bjørnetrø**: conceptualization, formal analysis, validation, investigation, visualization, methodology, writing – original draft, project administration, writing – review and editing. **Paula A. Bousquet**: conceptualization, formal analysis, validation, investigation, methodology, writing – review and editing. **Adriana M. Sanabria**: software, formal analysis, validation, investigation, methodology, writing – review and editing. **Sebastian Meltzer**: data curation, methodology, writing – review and editing. **Torben Lüders**: validation, investigation, writing – review and editing. **Anne‐Marie Siebke Trøseid**: formal analysis, validation, investigation, writing – review and editing. **Espen Stang**: formal analysis, validation, investigation, writing – review and editing. **Anne Negård**: formal analysis, validation, investigation, writing – review and editing. **Elin Agathe Frøyen**: formal analysis, validation, investigation, writing – review and editing. **Aida Kapic Lunder**: formal analysis, validation, investigation, writing – review and editing. **Lars Gustav Lyckander**: formal analysis, validation, investigation, writing – review and editing. **Hans Christian D. Aass**: formal analysis, validation, investigation, writing – review and editing. **Kathrine Røe Redalen**: data curation, funding acquisition, writing – review and editing. **Anne Hansen Ree**: resources, funding acquisition, visualization, methodology, writing – original draft, writing – review and editing.

## Conflicts of Interest

The authors declare no conflicts of interest

## Supporting information




**Supplementary Material**: jex270097‐sup‐0001‐SuppMat.docx

## Data Availability

The datasets used and analysed in the current study, including the sequence datasets in a safe data storage facility at Akershus University Hospital, are available from the corresponding author on reasonable request and in accordance with the General Data Protection Regulation of the European Union. Transfer of data or materials will require approval from the Data Privacy Officer at Akershus University Hospital and, on some occasions, from the Regional Committee for Medical and Health Research Ethics of South‐East Norway.
