# Supplementary material for: Dissemination of Mitochondrial DNA Variants: Looking at the ‘Bigger’ Picture of the Tumour Microenvironment in Rectal Cancer Patients
Source: J Extracell Biol. 2025 Oct 30;4(11):e70097. doi: 10.1002/jex2.70097 (PMC12575058; doi:10.1002/jex2.70097)
Supplement: Supplementary file 1 — Supplementary Material: jex270097‐sup‐0001‐SuppMat.docx [file JEX2-4-e70097-s001.docx]

**Supplementary file**

**Supplementary Table S1. Clinicopathological characteristics of the study patients.**

|  |  | ***n* (%)** |
| --- | --- | --- |
| Median age, years (range) |  | 63 (41-82) |
| Sex (*n* = 60) | Male | 41 (68.3) |
|  | Female | 19 (31.7) |
| T stage (*n* = 60) | 2 | 9 (15.0) |
|  | 3 | 27 (45.0) |
|  | 4 | 24 (40.0) |
| N stage (*n* = 60) | 0 | 25 (41.7) |
|  | 1 | 21 (35.0) |
|  | 2 | 14 (23.3) |
| M stage (*n* = 60) | 0 | 55 (91.7) |
|  | 1 | 5 (8.3) |
| Treatment (*n* = 60) | LC-CRT + surgery | 42 (70.0) |
|  | SCRT + surgery | 5 (8.3) |
|  | Only neoadjuvant | 2 (3.3) |
|  | Only surgery | 11 (18.3) |
| ypT stage (*n* = 49) | 0 | 8 (16.3) |
|  | 1 | 4 (8.2) |
|  | 2 | 5 (10.2) |
|  | 3 | 28 (57.1) |
|  | 4 | 2 (4.1) |
|  | ND* | 2 (4.1) |
| ypN stage (*n* = 49) | 0 | 29 (59.2) |
|  | 1 | 11 (22.4) |
|  | 2 | 7 (14.3) |
|  | ND* | 2 (4.1) |
| pT stage (*n* = 11) | 1 | 3 (27.3) |
|  | 2 | 0 |
|  | 3 | 6 (54.5) |
|  | 4 | 2 (18.2) |
| pN stage (*n* = 11) | 0 | 5 (45.5) |
|  | 1 | 6 (54.5) |
|  | 2 | 0 |

* Patient declined surgery.

*Abbreviations:* LC-CRT, long-course chemoradiotherapy; M, metastasis; N, node; ND, not determined; p, histopathologic status after surgery alone; SCRT, short-course radiotherapy; T, tumor; yp, histopathologic status after neoadjuvant oncologic treatment and surgery.

**Supplementary Table S2. Sequencing depth.**

|  | Sequencing depth | |
| --- | --- | --- |
| Patient ID | WB mtDNA | Plasma EV mtDNA |
| Patient 1 | 12809 | 4740 |
| Patient 2 | 12305 | 14488 |
| Patient 3 | 4450 | 15339 |
| Patient 4 | 13198 | 4807 |
| Patient 5 | 13434 | 12250 |
| Patient 6 | 14206 | 7579 |
| Patient 7 | 12521 | 8926 |
| Patient 8 | 9188 | 13162 |
| Patient 9 | 12007 | 9939 |
| Patient 10 | 5513 | 12752 |
| Patient 11 | 12909 | 12499 |
| Patient 12 | 11245 | 12137 |
| Patient 13 | 11264 | 10050 |
| Patient 14 | 14914 | 2521 |
| Patient 15 | 8433 | 11687 |
| Patient 16 | 11023 | 7444 |
| Patient 17 | 15521 | 9263 |
| Patient 18 | 15978 | 3168 |
| Patient 19 | 13566 | 2667 |
| Patient 20 | 12347 | 15745 |
| Patient 21 | 10385 | 16530 |
| Patient 22 | 12054 | 7645 |
| Patient 23 | 16164 | 5803 |
| Patient 24 | 13327 | 3515 |
| Patient 25 | 14163 | 15036 |
| Patient 26 | 20489 | 4769 |
| Patient 27 | 12025 | 15900 |
| Patient 28 | 12102 | 12445 |
| Patient 29 | 11551 | 10072 |
| Patient 30 | 10558 | 8482 |
| Patient 31 | 11263 | 4544 |
| Patient 32 | 13027 | 11951 |
| Patient 33 | 16757 | 16330 |
| Patient 34 | 18611 | 14955 |
| Patient 35 | 13926 | 3033 |
| Patient 36 | 13321 | 3199 |
| Patient 37 | 10180 | 12106 |
| Patient 38 | 14337 | 15890 |
| Patient 39 | 40737 | 15898 |
| Patient 40 | 13208 | 16131 |
| Patient 41 | 5108 | 2760 |
| Patient 42 | 3205 | 3427 |
| Patient 43 | 46193 | 2935 |
| Patient 44 | 12410 | 3049 |
| Patient 45 | 11889 | 15523 |
| Patient 46 | 14610 | 16938 |
| Patient 47 | 14473 | 14153 |
| Patient 48 | 13117 | 15666 |
| Patient 49 | 13805 | 15242 |
| Patient 50 | 10051 | 15966 |
| Patient 51 | 11187 | 35950 |
| Patient 52 | 13699 | 18068 |
| Patient 53 | 12326 | 15079 |
| Patient 54 | 12610 | 19981 |
| Patient 55 | 13529 | 6792 |
| Patient 56 | 19797 | 12440 |
| Patient 57 | 13160 | 15074 |
| Patient 58 | 10354 | 10885 |
| Patient 59 | 14049 | 17841 |
| Patient 60 | 13239 | 15080 |

**Supplementary Figure S1.**

Mitochondrial DNA in paired samples of whole blood (WB) and isolated B-cells, CD4 T-cells, CD8 T-cells, and monocytes from rectal cancer patients (*n* = 5). The mitochondrial DNA **A,** total variant number (TVN) and **B,** heteroplasmic variant number (HVN; frequency <0.990). Comparisons were calculated with a repeated measures ANOVA with Turkey’s multiple comparison test (ns, not significant).

One to two million frozen peripheral blood mononuclear cells were thawed, washed, and stained in RPMI-1640 medium with 10 ng/mL anti-CD45 V450 (catalog no. 642275, BD Biosciences), 20 ng/mL anti-CD19 APC-H7 (catalog no. 0314401, BD Biosciences), 10 ng/mL anti-CD3 FITC (catalog no. 0342661, BD Biosciences), 3 ng/mL anti-CD8 APC (catalog no. 555369, BD Biosciences), 5 ng/mL anti-CD4 PE (catalog no. 566910, BD Biosciences), and 10 ng/mL anti-CD14 PE-Cy7 (catalog no. 557742, BD Biosciences). Cells were kept on ice, and fluorescence-minus-one controls were prepared to adjust gates to sort CD19 B-cells, CD8 and CD4 T-cells, and CD14 monocytes on a BD FACSAria cell sorter (BD Biosciences). Sorted populations were reanalyzed and purity was determined (>98%). Cells were immediately frozen for further downstream DNA isolation from an average of 136,900 cells (range 3500-434,000).

**Supplementary Figure S2.**

Plasma extracellular vesicles (EVs) size distribution as assessed by transmission electron microscopy and Nanoparticle Tracking Analysis in two study patients. Blue: EVs from a patient with small vesicle mode size (96.8 nm). Red: EVs from a patient with larger vesicle mode size (114.0 nm). The position of the zoom within the wide-field view is indicated by a black box; scale bars are 500 nm (black lines) and 200 nm (dotted lines).
